# ADAMTS-1 in abdominal aortic aneurysm

**DOI:** 10.1371/journal.pone.0178729

**Published:** 2017-06-01

**Authors:** Emina Vorkapic, Maggie Folkesson, Kerstin Magnell, Mohammad Bohlooly-Y, Toste Länne, Dick Wågsäter

**Affiliations:** 1 Division of Drug Research, Department of Medical and Health Sciences, Linköping University, Linköping, Sweden; 2 Division of Discovery Sciences, Innovative Medicines, AstraZeneca R&D, Mölndal, Sweden; 3 Division of Cardiovascular Medicine, Department of Medical and Health Sciences, Faculty of Health Sciences, Linköping University, Linköping, Sweden; 4 Department of Cardiovascular Surgery, County Council of Östergötland, Linköping, Sweden; Max Delbruck Centrum fur Molekulare Medizin Berlin Buch, GERMANY

## Abstract

**Introduction:**

Extracellular matrix degradation is a hallmark of abdominal aortic aneurysm (AAA). Among proteases that are capable of degrading extracellular matrix are a disintegrin and metalloproteases with thrombospondin motifs (ADAMTS). Pathogenesis of these proteases in AAA has not been investigated until date.

**Methods and results:**

Human aneurysmal and control aortas were collected and analyzed with RT-PCR measuring the ADAMTS-1, 4,5,6,8,9,10,13,17 and ADAMTSL-1. Expression of a majority of the investigated ADAMTS members on mRNA level was decreased in aneurysm compared to control aorta. ADAMTS-1 was one of the members that was reduced most. Protein analysis using immunohistochemistry and western blot for localization and expression of ADAMTS-1 revealed that ADAMTS-1 was present predominantly in areas of SMCs and macrophages in aneurysmal aorta and higher expressed in AAA compared to control aortas. The role of ADAMTS-1 in AAA disease was further examined using ADAMTS-1 transgenic/*apoE*^*-/-*^ mice with the experimental angiotensin II induced aneurysmal model. Transgenic mice overexpressing ADAMTS-1 showed to be similar to ADAMTS-1 wild type mice pertaining collagen, elastin content and aortic diameter.

**Conclusion:**

Several of the ADAMTS members, and especially ADAMTS-1, are down regulated at mRNA level in AAA, due to unknown mechanisms, at the same time ADAMTS-1 protein is induced. The cleavage of its substrates, don’t seem to be crucial for the pathogenesis of AAA but rather more important in the development of thoracic aortic aneurysm and atherosclerosis as shown in previous studies.

## Introduction

Numerous groups of proteases have been suggested to be involved in the remodelling of the extracellular matrix. The most studied family of matrix-degrading enzymes, which we also contributed to, is probably the matrix metalloproteinases (MMPs) since these proteases are capable to degrade plenty of macromolecules present in the connective tissue matrix [1–3]. The extensive family of matrix metalloproteases beside MMPs includes a disintegrin and metalloproteinase (ADAMs) of which several members have been found in tissue from abdominal aortic aneurysms (AAA) [4, 5].

Closely related to the ADAM family are a disintegrin and metalloproteinase with thrombospondin motifs, (ADAMTS). ADAMTSs are secreted into the extracellular domain and some members of this family, in contrast to other metalloproteases, have capacity to bind to extracellular matrix [6]. ADAMTS similar to other metalloproteases contain a pro-domain that preserves ADAMTSs latency. Not all the substrates for ADAMTSs are known but they usually have various matrix proteins as substrate [7].

ADAMTS-1 is highly expressed in atherosclerotic aorta and promotes atherogenesis by cleaving extracellular matrix proteins such as versican and inducing vascular smooth muscle cell migration [8]. We previously found that ADAMTS-4 and -8 are inflammatory regulated enzymes expressed in LDLR^-/-^/apoB^100/100^ mice and in macrophage-rich areas of human atherosclerotic carotid plaques and coronary unstable plaques [9].

AAA occurs when extracellular matrix of the aorta has degenerated, and the aortic diameter enlarges irreversibly with a deadly outcome in the form of rupture. None of the currently known members of ADAMTS family have been studied in relation to AAA. In this study we focused our investigation on the presence and expression of ADAMTS-1. We also included mRNA expression analysis of several other members of our interest such as 4, 5, 6, 8, 9, 10, 13, 17 and ADAMTSL-1 in the aortic wall of control aortas and AAA. The role of ADAMTS-1 in AAA was further investigated using angiotensin II induced AAA in mice.

Our data evaluating the expression of ADAMTS members in human aneurysm shows that mRNA expression of several of the members are decreased in AAA samples as compared to control aortas. Mice overexpressing ADAMTS-1 in an angiotensin II induced AAA develop aneurysm to the same extent as wild type mice and therefore ADAMTS-1 probably plays minor role in AAA progression.

## Materials and methods

### Human samples

AAA patients (n = 16, all men with mean age of 66, range 56–74 with aortic diameter of 65 mm, range 47–75) undergoing surgical repair were included in this study and biopsies from thrombus-free (NTH, n = 12) and thrombus-covered (TH, n = 16) aneurysm wall were taken since previous studies have revealed major differences in inflammatory response between aneurysm walls covered and not covered by an intraluminal thrombus [2]. Samples included were sufficient to detect a difference of two-fold with 95% confidence interval and at >80% power. Aortic walls were assayed as whole aorta and part of them were dissected to adventitia and media in aneurysmal aorta. Aorta from organ donors (n = 13, all men with mean age of 49, range 32–61) without clinical or macroscopic signs of aortic atherosclerosis or aneurysm were used as non-aneurysmal external controls. Biopsies were immediately fixed in 4% formaldehyde for histology analyses, snap frozen in liquid nitrogen for protein analysis or placed in RNAlater (Ambion, Austin, TX, USA) overnight in 4′C and then stored in -80′C for RNA isolation.

All AAA participants gave written informed consent to the study, which was approved by the regional ethical review board in Linköping, Sweden. The use of organ donors was approved by the regional ethical review board in Lund, Sweden.

### AngII-induced AAA in mice

AngII-induced aneurysm is an inflammation-driven model that is frequently used to experimentally induce AAA [10–12]. Aneurysm was induced in wild type male hypercholesterolemic *apoE*^*-/-*^ mice (Taconic, Bomholt, Denmark) mice (n = 12) and *apoE*^*-/-*^*/ADAMTS-1* transgenic mice (n = 14), established and obtained from Astrazeneca, Gothenburg, Sweden as previously described [8]. Animals included were sufficient to detect a 20% difference with 95% confidence interval at >80% power. Animals were housed in groups in pathogen free (SPF) facility in IVC enriched cages and maintained on a 12:12 hour light/dark cycle.

At eight weeks of age, AAA was induced, in randomly chosen mice, by chronic infusion of 1000 ng/kg/min AngII (Cat.no.9525, Sigma Aldrich, St. Louis, USA) via mini-osmotic pumps (Model 1004, Alzet, CA, USA) as described previously [11]. Pumps were placed under anesthesia using isoflurane. Post-operative pain was treated with buprenorphine at a dose of 0.1 mg/kg body weight, injected subcutaneously twice per day for 3 days. A group of *apoE*^*-/-*^ mice were infused with 0.9% NaCl and were used as control mice. Standard Chow diet and water was allowed *ad libidum* throughout the whole study and mice were monitored daily for signs of discomfort. After 28 days, mice were sacrificed. The aorta was removed and fixated in RNAlater for 24 hours thereafter frozen in -70°C for gene expression analysis or snap frozen for protein analysis. Part of aorta was fixed in formaldehyde and embedded in paraffin for histology and analysis of aneurysm formation. The study was approved by the local ethical committee in Linköping, Sweden (47–13).

### Immunohistochemistry

Paraffin-embedded human abdominal aortas from four patients were sectioned (5 μm) and rehydrated in several changes of ethanol and Tissue-Clear^®^ (Sakura Finetek, Leiden, The Netherlands). Endogenous peroxidase activity was quenched by treatment with 3% hydrogen peroxide for 5 min followed by incubation in 5% blocking rabbit or goat serum albumin solution. Sections were then incubated with human primary antibodies against ADAMTS-1 (2 μg/ml, Cat. No. AF5867, R&D Systems, Inc, Minneapolis, USA), CD68 (0.1 μg/ml, Cat. No. NCL-CD68-KP1, Leica Microsystems, Newcastle, UK) and α-actin (0.6 mg/ml, cat. No. A5228, clone 1A4, Sigma-Aldrich) at 4°C overnight followed by secondary biotinylated rabbit anti-sheep IgG or goat anti-mouse IgG antibody (Dako, Stockholm, Sweden). Avidin-biotin peroxidase complexes (Dako) were added followed by visualization with 3,3′-diaminobenzidine tetrahydrochloride (Dako). All sections were counterstained with Mayer’s hematoxylin (Histolab Products, Göteborg, Sweden).

### Western blot

Frozen aortas from AAA patients and non-aneurysmal control donors were homogenised in ice-cold RIPA lysis buffer containing 50 mM Tris-HCl pH 7.4, 150 mM NaCl, 1 mM EDTA, 0.25% Na-deoxy-cholate, 1% Triton-100 and 0.1% SDS, 100 μg/ml phenylmethylsulphonyl fluoride (PMSF) and protease inhibitor cocktail (Roche Diagnostics Scandinavia, Stockholm, Sweden). Protein quantification of tissue extracts (30 μg of protein) were separated under reducing conditions using 4–12% gradient SDS-PAGE and transferred onto a polyvinylidine difluoride membrane (Amersham Life Science, Amersham, UK). Membranes were blocked in 5% non-fat dry milk and incubation with primary rabbit ADAMTS-1 antibody (1 μg/ml, Cat. No. AF5867, R&D Systems) overnight at 4°C followed by incubation by a horseradish peroxidase-conjugated secondary anti-sheep antibody (1:5000, Cat. No. sc-2770, Santa Cruz Biotechnology, Heidelberg, Germany). Immunoreactive proteins were visualized by chemiluminescence ECL Prime Western blotting detection system (Amersham). Loading was controlled using coomassie blue staining of membranes.

### Elastin and collagen staining

Paraffin-embedded sections (5 μm) from mice aorta were rehydrated in several changes of ethanol and Tissue-Clear^®^ (Sakura Finetek). Sections were then stained for collagen and elastin as previously described [13]. Sections were studied under light microscopy ZEN 2012 (Zeiss, Jena, Germany). Quantification of collagen and elastin were performed blinded. A scoring system from 1 to 4 was used with 1 defined as no degradation of elastin, 2 as low degradation, 3 as intermediate degradation, and 4 as high degradation. For collagen quantification, 1 defined as low amount of collagen in all layers of the aorta, 2 as low amount of collagen in aorta media or in aorta adventitia, 3 as high amount of collagen in aorta media or in aorta adventitia, and 4 as high amount of collagen in all layers of the aorta. The suprarenal aortic diameter was measured as the adventitial diameter and the definition of an aneurysm was set as a 1.5-fold enlargement of leading edge to leading edge. Measurements of the aortic diameter was performed using Zen 2012 (blue edition, Carl Zeiss Microscopy GmbH, 2011 ver 1.1.2.0, Oberkochen, Germany). To measure aortic outer diameter, the largest dilated part of the aorta was taken for this purpose.

### Quantitative real-time PCR

Human and mouse aortas were homogenized with trizol and chloroform in a Tissue Lyser using safe-lock tubes with a metal bead. Total RNA from human and mice aortas were isolated with RNeasy mini kit (Qiagen, Hilden, Germany) and reversely transcribed with random primers and Superscript III (Invitrogen, Carlsbad, USA) according to manufacturer′s instructions. Quantitative real-time polymerase chain reaction (PCR) was performed on ABI 7500 Fast real-time PCR Sequence Detector using gene expression assay primers and 2x TaqMan Fast Universal PCR Mastermix (Applied Biosystems, Foster City, USA). Amplified cDNA (human 0.25 μg; mouse 0.126 μg) was run in duplicates. All probes were obtained from Applied Biosystems and the results were normalized to values of human RPLP0 or mouse Gapdh. Human ADAMTS1, 4, 5, 6, 8, 9, 10, 13, 17 and ADAMTSL-1 and mouse ADAMTS1 were analyzed.

### Statistical analysis

The statistical analysis was performed with the IMB SPSS Statistics 22. Comparisons of quantitative data were performed using Student′s t-test and one way analysis of variance (ANOVA). Multiple test correction was performed using Bonferroni post hoc analysis. P-values < 0.05 were considered statistically significant where * indicates P < 0.05, ** indicates P < 0.01 and *** indicates P < 0.001.

## Results and discussion

### mRNA expression of ADAMTS members in AAA

**Fig 1:**
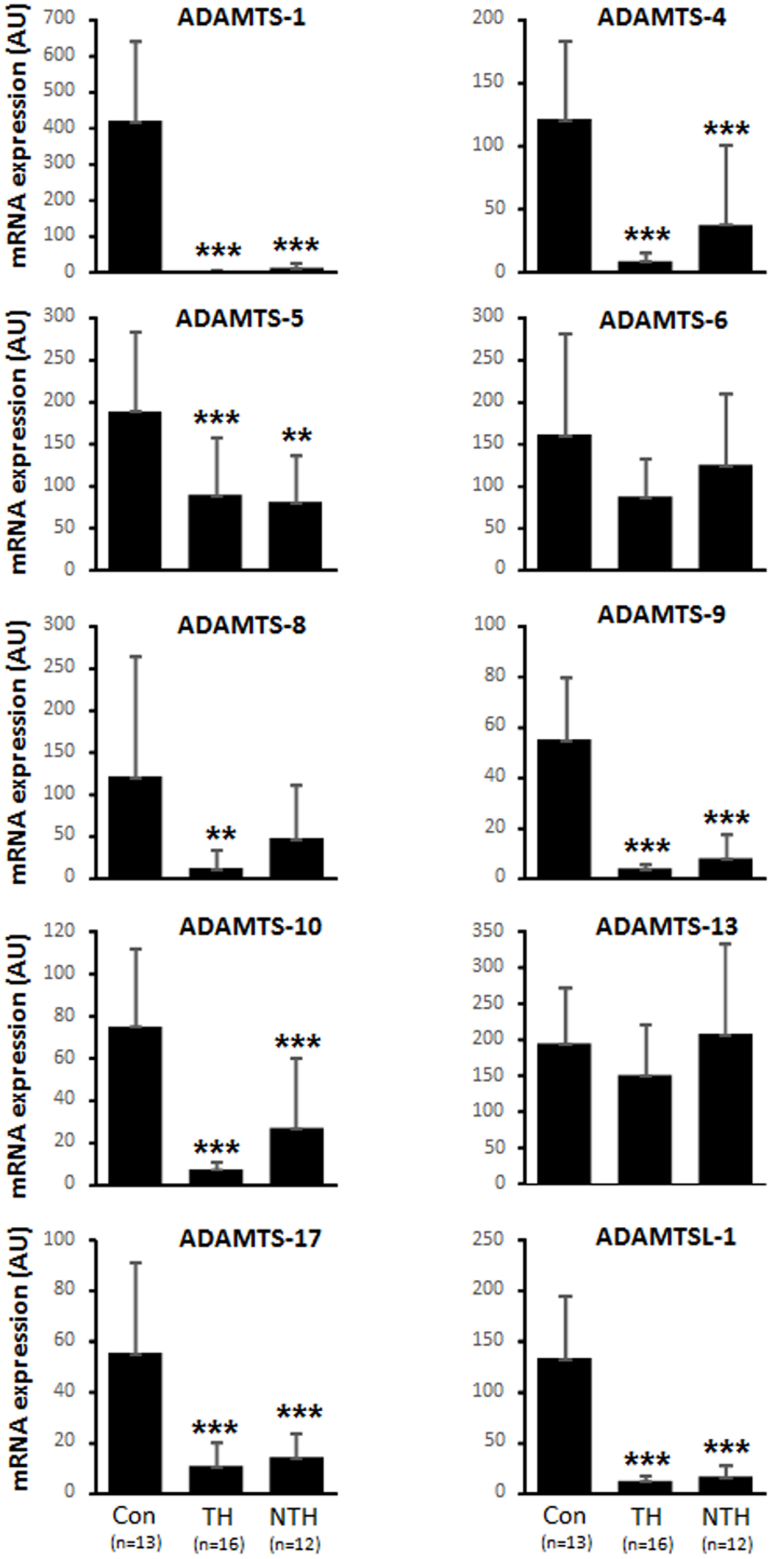
Gene expression of ADAMTS members. Expression in non-aneurysmal control (con) aortas and aneurysmal aorta covered with intraluminal thrombus (TH) and not covered with intraluminal thrombus (NTH). * p<0.05; ** p<0.01; *** p<0.001 v.s. control using one way ANOVA with Bonferroni post hoc analysis.

mRNA expression of nine of *ADAMTS-1*, *4*, *5*, *6*, *8*, *9*, *10*, *13*, *17* and ADAMTSL-1, were analyzed in aortic wall of aneurysm patients and control aortas using real time RT-PCR (Fig 1). These data show that most of the members are down regulated on mRNA level in aneurysmal aorta compared to control aorta. ADAMTS-1 was one of the family members with the largest reduction reaching a 99% reduction in the expression in the vessel wall under the thrombus and 97% reduction in thrombus free vessel wall. The reduction was more prominent in the intima/media layer compared to the adventitia that showed an 80% higher expression in ADAMTS-1 (S1 Fig).

Aneurysmal aorta under intraluminal thrombus is severely distorted compared to the aneurysmal wall that is free from thrombus. The lack of extracellular matrix could possibly cause the disappearance of ADAMTSs proteases since they bind to extracellular macroproteins. Our and others previous data indicate that downregulated factors on mRNA level may reflect the phenotypic state of the SMC or the fact that the protein is already produced in high levels. In response to injury, SMC undergo phenotypic modulation into the synthetic SMC, exhibiting a rapid increase in proliferation, migration and production of ECM components. The presence of synthetic SMC is characterized by loss of the cell surface markers that are associated with the normal state contractile SMC [14]. However, normalizing the data to SMC markers α-actin and SM22α did not influence the results (data not shown). A limitation of the current study is the mismatch in age between controls and patients, which is difficult to control for in control subjects. However, adjustment for age did not change the result and in the mice experiments, the groups are age matched which showed similar pattern in respect to ADAMTS-1 mRNA expression between control and aneurysmal aortas.

### Protein expression and localization of ADAMTS-1 in AAA

**Fig 2:**
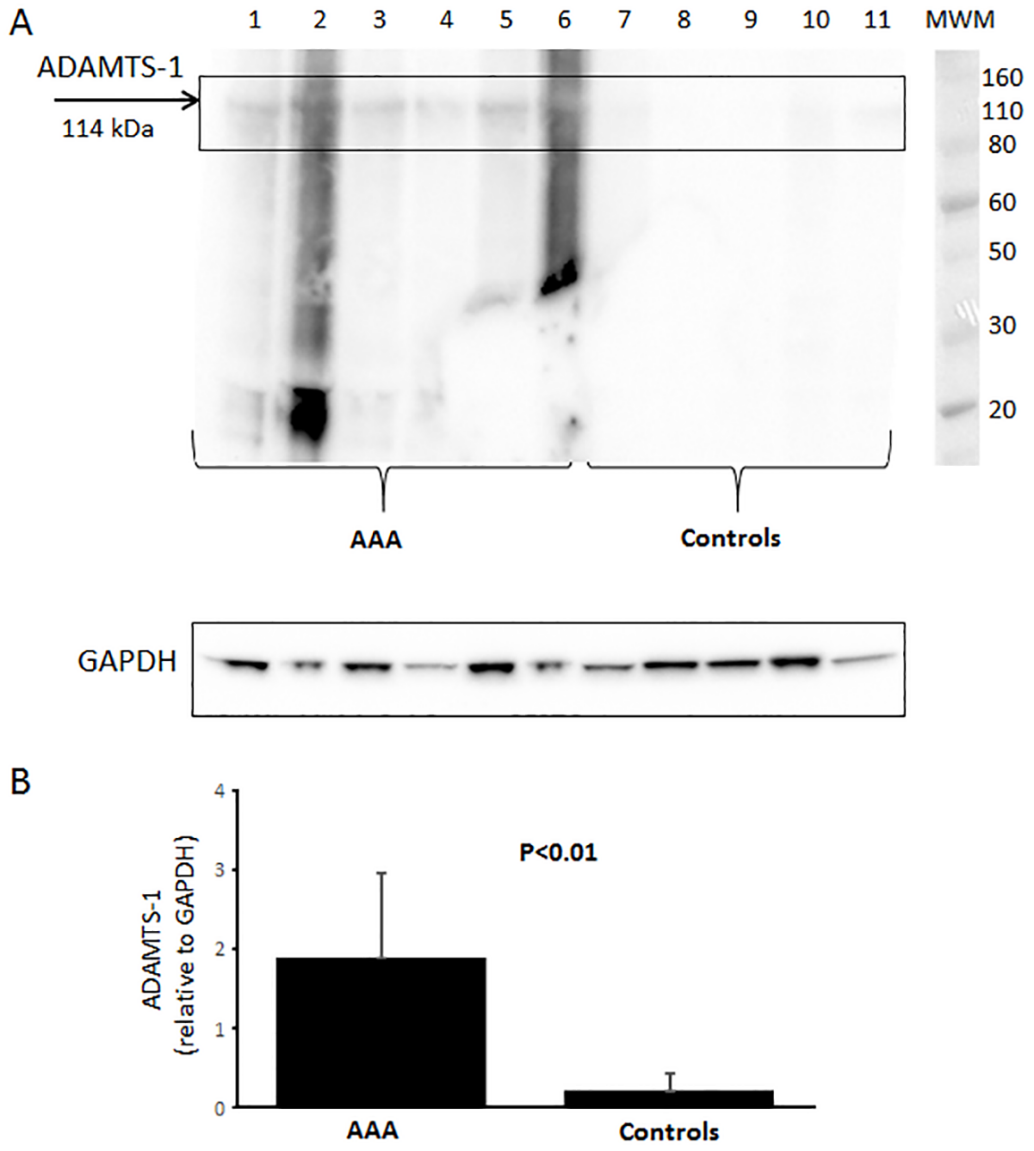
Western blot analysis. (A) Expression of ADAMTS-1 and GAPDH in human AAA aortas (sample 1–6) and human non-aneurysmal control aortas (sample 7–11) and (B) quantification of ADAMTS-1 in relative values normalized to GAPDH. MWM: molecular weight marker indicating size in kDa.

ADAMTS-1 was one of the family members with the largest changes in respect to mRNA expression and taken forward for protein analysis using immunohistochemistry and western blot. The western blot results showed a weak expression of ADAMTS-1 in human control aorta compared to a much stronger expression in aneurysmal aorta (Fig 2). The stronger smears in lanes 2 and 6 and bands at lower molecular weight comes from the secondary antibody (data not shown).

**Fig 3:**
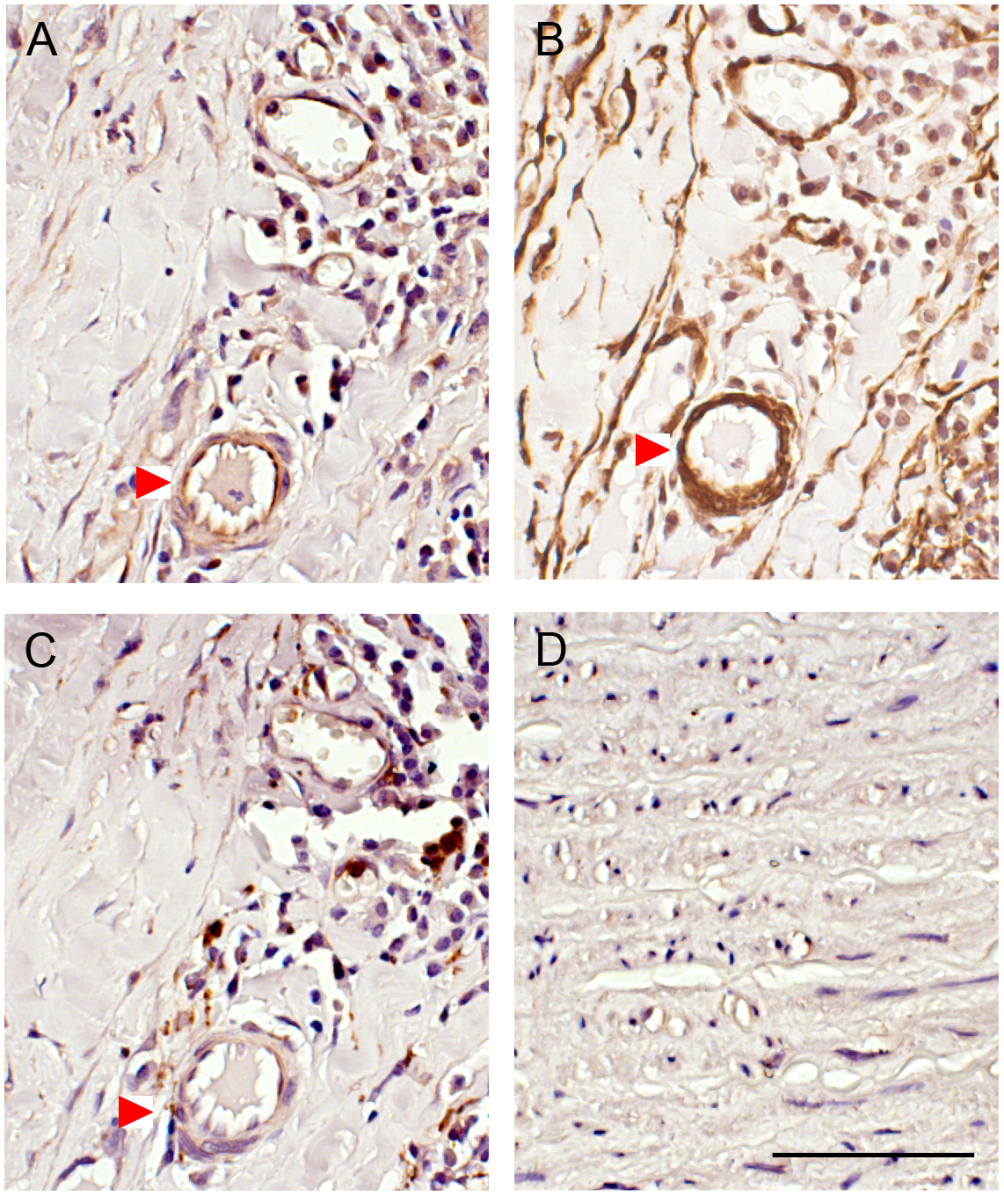
Immunohistochemical staining. (A) ADAMTS-1, (B) SMC marker α-actin, (C) macrophage marker in adventitial layer of human AAA aortas and (D) ADAMTS-1 in human non-aneurysmal control aortas. Staining of positive cells are seen in brown. The images are taken with 40X magnification and scale bar represent 50 μm. Red arrow indicates the same area.

This increase was confirmed with the immunohistochemical analysis (Fig 3). From this it was observed that the primary source of ADAMTS-1 in AAA are adjacent to smooth muscle cells in adventitia (Fig 3A and 3B) and media layer (data not shown) and to some degree around areas of macrophages present in the aneurysmal wall (Fig 3C). Normal aorta (Fig 3D) contains orderly organized layers of intima, media and adventitia with relatively low protein expression of ADAMTS-1. However the distorted aneurysmal wall lacks this pattern due to apoptosis of smooth muscle cell, fragmentation of elastin, overproduction of collagen as a protective mechanism and presence of inflammatory cells throughout the wall with no distinctive aortic layout.

Expression of ADAMTS-1 and -4 and its substrate versican have previously been examined in thoracic aortic aneurysm [15]. ADAMTS-1 and ADAMTS-4 protein and mRNA expression was significantly higher in thoracic aortic aneurysm and dissection tissues with a concomitant degradation of versican. In concordance with our results, ADAMTS-1 was localized to smooth muscle cells and macrophages in the thoracic aortic aneurysm. These results indicates differences in transcriptional regulation of ADAMTS members in thoracic and abdominal aortic aneurysm.

### Possible effects of ADAMTS-1 in experimentally induced AAA in mice

**Fig 4:**
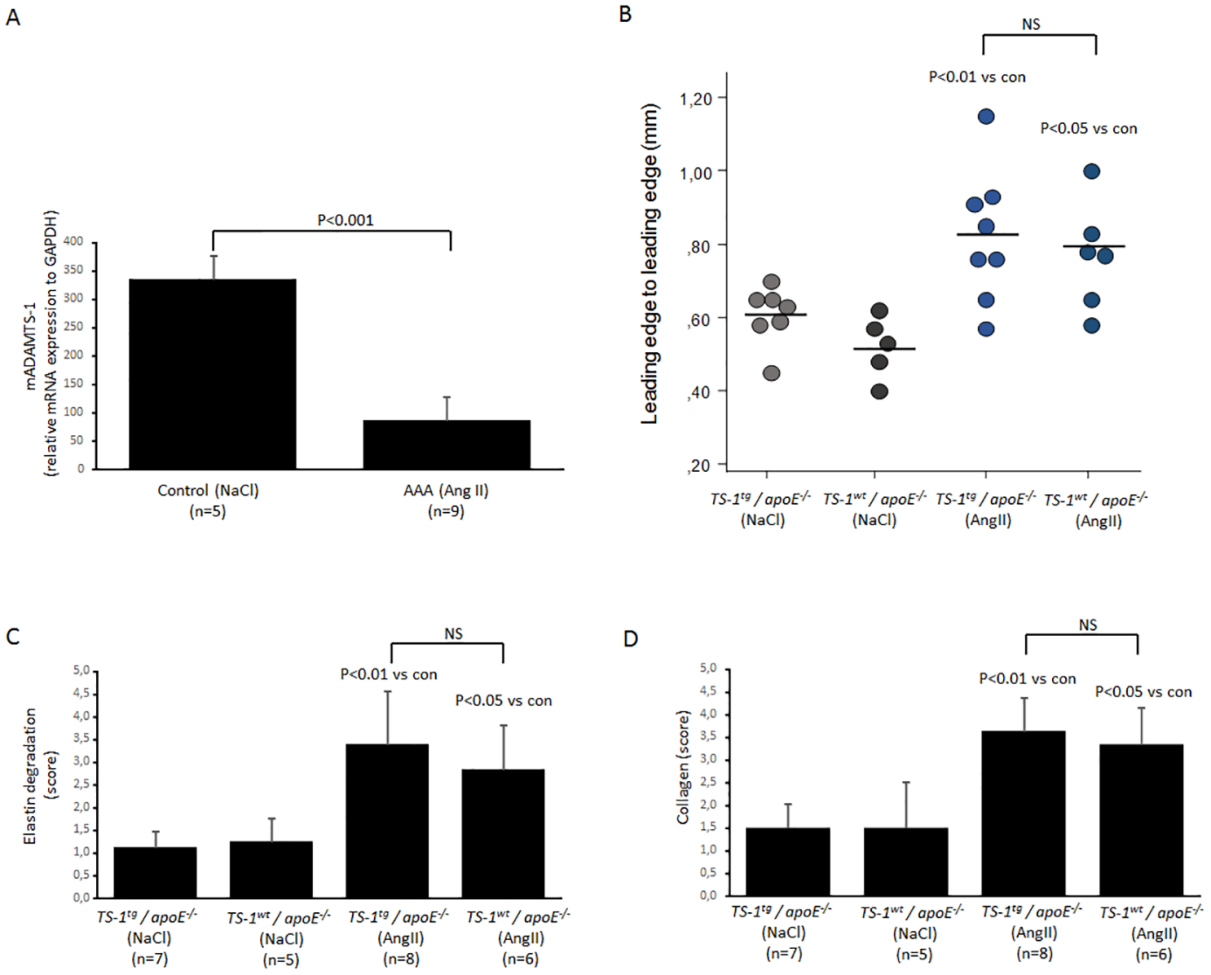
AngII induced AAA. (A) Gene expression of ADAMTS-1 in mouse aortas. (B) ADAMTS-1 wild type (wt) and ADAMTS-1 transgenic mice (tg) induced with AngII show enlarged aortic diameter but with no significant difference. (C) These enlargements are largely dependent on loss of elastin and (D) a compensatory collagen overproduction but with no significant differences between the groups. 1–2 sections per mouse were analyzed. Student t-test was used in panel A and ANOVA with Bonferroni post hoc analysis in panel B-D.

Aneurysm was induced in mice using the established angII model. Similar to human aneurysm, aneurysm in mice caused a significant reduction in expression of *ADAMTS-1* mRNA in their aorta (Fig 4A).

To investigate how ADAMTS-1 could affect AAA formation we used transgenic mice that overexpress ADAMTS-1 and induced aneurysm in these mice along with control mice (ADAMTS-1^wt^). Overexpression of ADAMTS-1 did not affect aneurysm formation in these mice, in other words we did not observe any significant difference in aortic diameter or aneurysm incidence between ADAMTS-1 transgenic and wild type mice (Fig 4B). Incidence of aneurysm was 50% in both angII induced groups (4/8 in the transgenic and 3/6 in the corresponding controls) as determined by aortic diameter as well as macroscopic and microscopic signs. This is in line with our and others previous results and in respect to 3R we did decide to not include more animals. Scoring amount of degraded elastin and total collagen deposited in the aortas, the mice with aneurysm showed a significant difference with controls in the form of vast elastin degradation and collagen deposition but there was again no difference between the ADAMTS-1 transgenic and ADAMTS-1 wild type pertaining degraded elastin fibers and collagen content (Fig 4C and 4D). Example of Masson trichrome staining is found in S2 Fig. From these experiments we conclude that the content of collagen and aneurysm formation in aorta in mice overexpressing ADAMTS-1 was not affected.

## Conclusion

Several of the ADAMTS members, and especially ADAMTS-1, are down regulated at mRNA level in AAA, due to unknown mechanisms, at the same time ADAMTS-1 protein is induced. The cleavage of its substrates, therefore don’t seem to be crucial for the pathogenesis of AAA but rather more important in the development of thoracic aortic aneurysm and atherosclerosis as shown in previous studies. To further elucidate any eventual role of ADAMTS-1 in aneurysm formation, other aneurysm models such as the CaCl_2_ and the elastase model and knock outs of ADAMTS-1 needs to be performed since the limitation of this study is the inclusion of only one experimental model.

## Supporting information

S1 Fig Gene expression of ADAMTS-1 in human aneurysm. Gene expression of ADAMTS-1 in aneurysmal aorta covered with intraluminal thrombus (TH) and not covered with intraluminal thrombus (NTH) divided into adventitia (adv) or intima/media (med) layer. P<0.05 using Student t-test.
(PPTX)

S2 Fig Masson trichrome staining. ADAMTS-1 (wt) x *apoE*^*-/-*^ with NaCl (A) or angII infusion (B) ADAMTS-1 (tg) x *apoE*^*-/-*^ in NaCl (C) and AngII infusion (D). Collagen in blue and smooth muscle cells in red. Aneurysm development in AngII mice with compensatory collagen production is seen in both ADAMTS-1 transgenic and wild type *apoE*^*-/-*^ mouse. Scale bar represents 50 μm.
(PPTX)
