# Supplementary material for: ADAMTS-1 in abdominal aortic aneurysm
Source: PLoS One. 2017 Jun 1;12(6):e0178729. doi: 10.1371/journal.pone.0178729 (PMC5453572; doi:10.1371/journal.pone.0178729)
Supplement: S1 Fig — Gene expression of ADAMTS-1 in aneurysmal aorta covered with intraluminal thrombus (TH) and not covered with intraluminal thrombus (NTH) divided into adventitia (adv) or intima/media (med) layer. P<0.05 using Student t-test. (PPTX) [file pone.0178729.s001.pptx]

## Slide 1
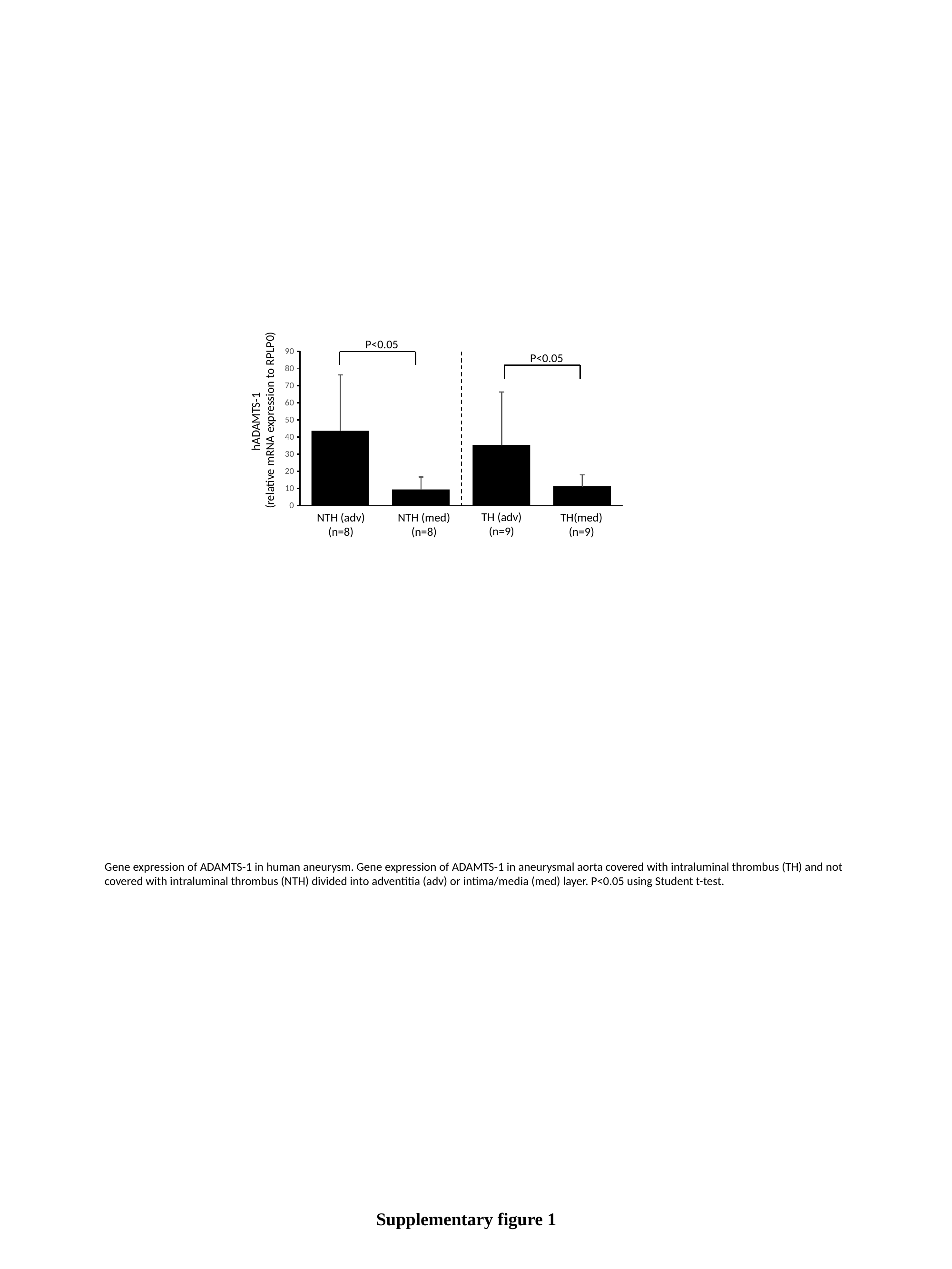

P<0.05
90
P<0.05
80
70
60
hADAMTS-1
(relative mRNA expression to RPLP0)
50
40
30
20
10
0
TH (adv)
(n=9)
NTH (adv)
(n=8)
NTH (med)
(n=8)
TH(med)
(n=9)
Gene expression of ADAMTS-1 in human aneurysm. Gene expression of ADAMTS-1 in aneurysmal aorta covered with intraluminal thrombus (TH) and not covered with intraluminal thrombus (NTH) divided into adventitia (adv) or intima/media (med) layer. P<0.05 using Student t-test.
Supplementary figure 1
