# Supplementary material for: ADAMTS-1 in abdominal aortic aneurysm
Source: PLoS One. 2017 Jun 1;12(6):e0178729. doi: 10.1371/journal.pone.0178729 (PMC5453572; doi:10.1371/journal.pone.0178729)
Supplement: S2 Fig — ADAMTS-1 (wt) x apoE-/- with NaCl (A) or angII infusion (B) ADAMTS-1 (tg) x apoE-/- in NaCl (C) and AngII infusion (D). Collagen in blue and smooth muscle cells in red. Aneurysm development in AngII mice with compensatory collagen production is seen in both ADAMTS-1 transgenic and wild type apoE-/- mouse. Scale bar represents 50 μm. (PPTX) [file pone.0178729.s002.pptx]

## Slide 1
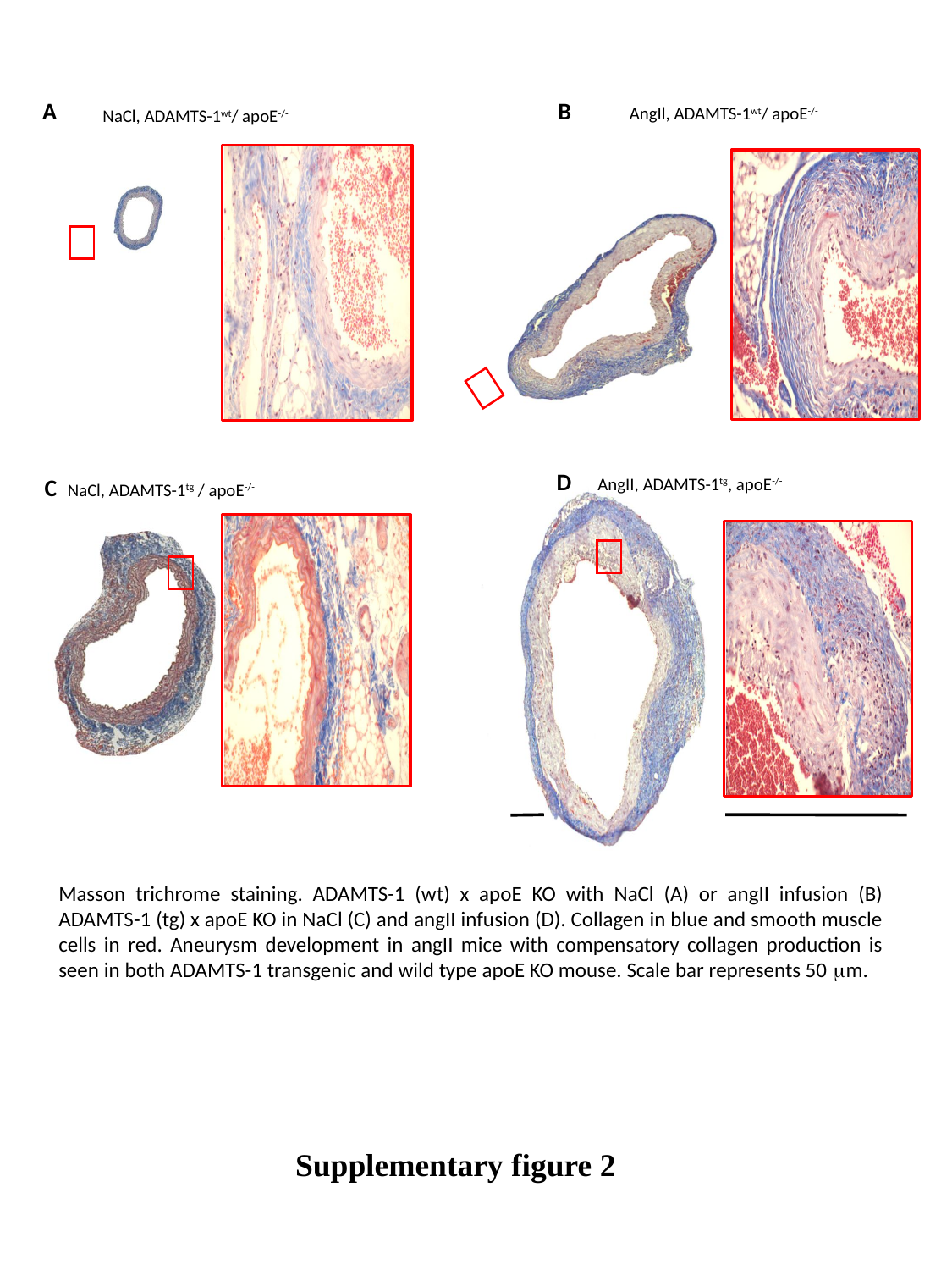

A
B AngIl, ADAMTS-1wt/ apoE-/-
 NaCl, ADAMTS-1wt/ apoE-/-
D AngII, ADAMTS-1tg, apoE-/-
C NaCl, ADAMTS-1tg / apoE-/-
Masson trichrome staining. ADAMTS-1 (wt) x apoE KO with NaCl (A) or angII infusion (B) ADAMTS-1 (tg) x apoE KO in NaCl (C) and angII infusion (D). Collagen in blue and smooth muscle cells in red. Aneurysm development in angII mice with compensatory collagen production is seen in both ADAMTS-1 transgenic and wild type apoE KO mouse. Scale bar represents 50 μm.
Supplementary figure 2
